# Supplementary material for: Enhanced NO2 Sensing Performance of ZnO-SnO2 Heterojunction Derived from Metal-Organic Frameworks
Source: Nanomaterials (Basel). 2022 Oct 23;12(21):3726. doi: 10.3390/nano12213726 (PMC9658193; doi:10.3390/nano12213726)
Supplement: Supplementary file 1 [file nanomaterials-12-03726-s001.zip › nanomaterials-1971457-supplementary.pdf]

Supporting information

Humidity test: To investigate the NO_2 sensing performance under humidity environment, the target gas was humidified by passing through a quartz flask filled with saturated solutions of K_2CO_3 and pure deionized water to attain the relative humidity (RH) levels of 43%±2% and 90%±2%, respectively. Relative humidity and temperature of gas flow were detected by Intelligent Humi/Temp Data Logger produced by YOWEXA Sensor System CO., LTD. The relative humidity and temperature probe was fixed at the outlet of the quartz flask to measure the relative humidity and temperature of gas flow.

Table S1. EDS element result of S2.

Element	Weight%	Atomic%
O K	28.80	66.39
Zn L	45.30	25.56
Sn L	25.90	8.05
Totals	100.00	

Table S2. EDS element result of S3.

Element	Weight%	Atomic%
O K	32.69	75.44
Zn L	14.28	8.06
Sn L	53.03	16.49
Totals	100.00	

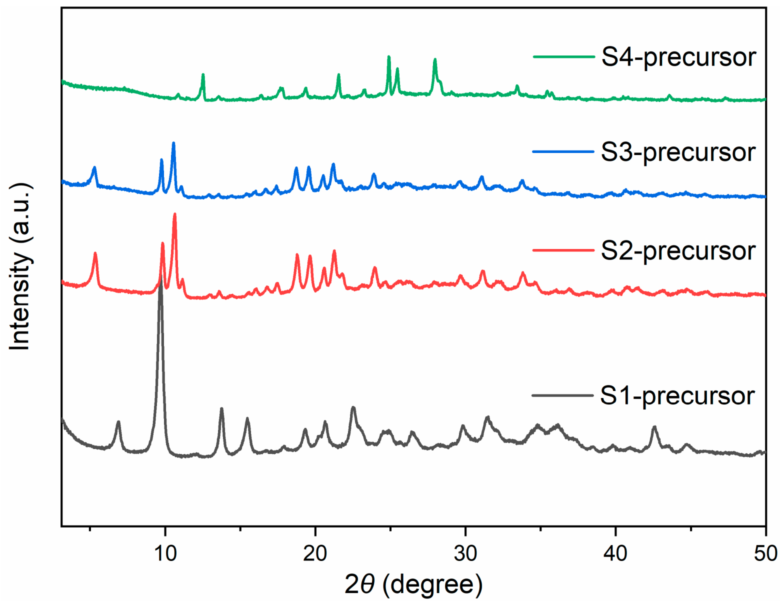

Figure S1. XRD of precursor.

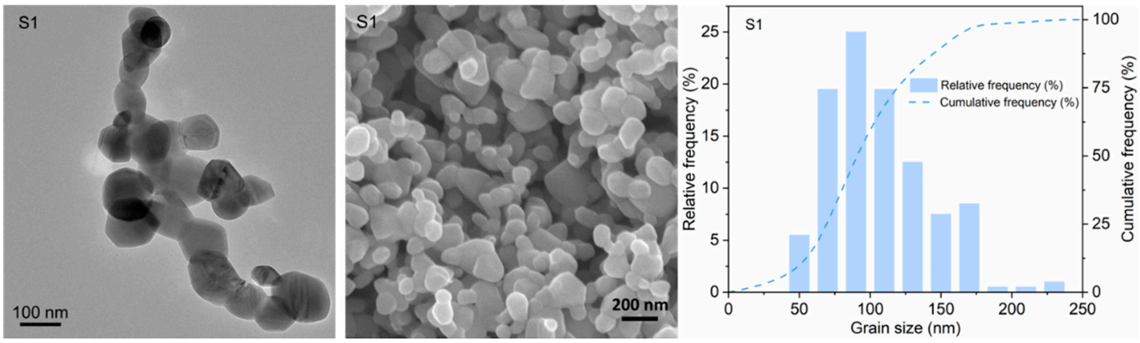

Figure S2. TEM, SEM images and corresponding grain size analysis of S1.

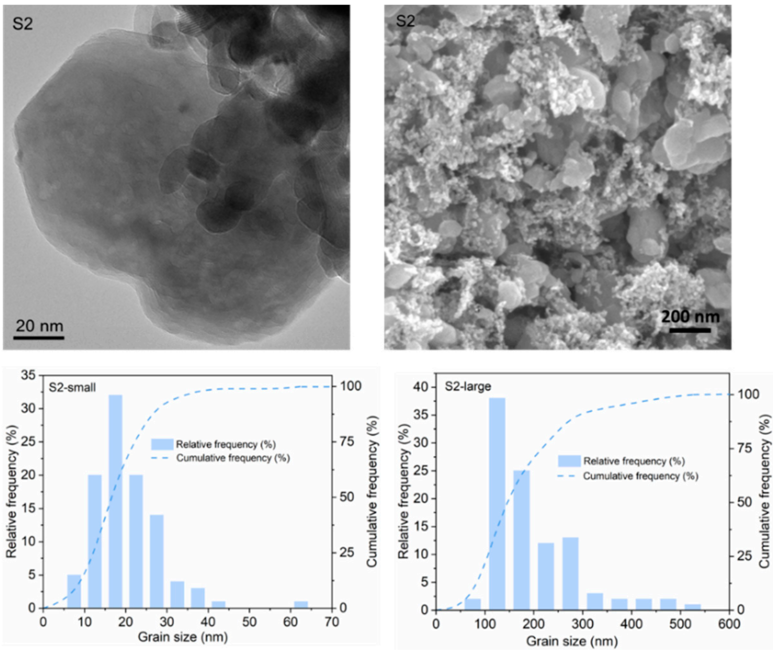

Figure S3. TEM, SEM images and corresponding grain size analysis of S2.

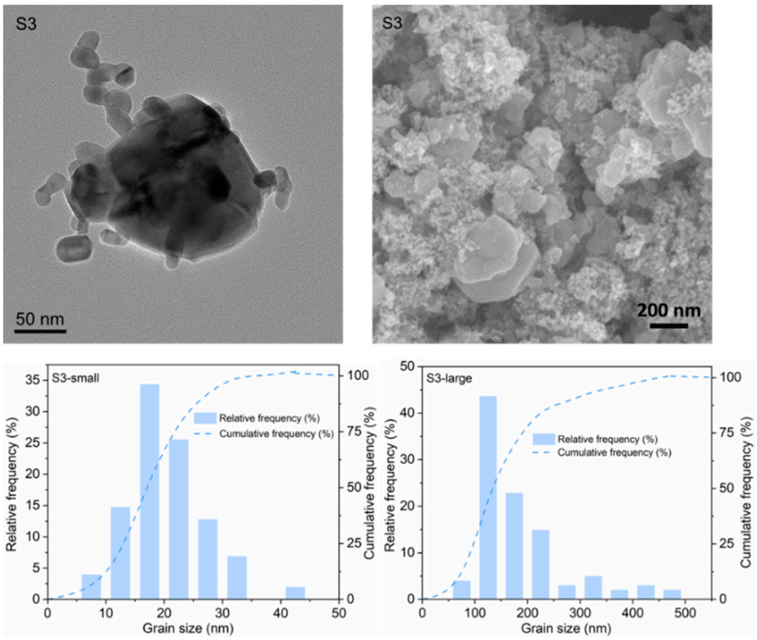

Figure S4. TEM, SEM images and corresponding grain size analysis of S3.

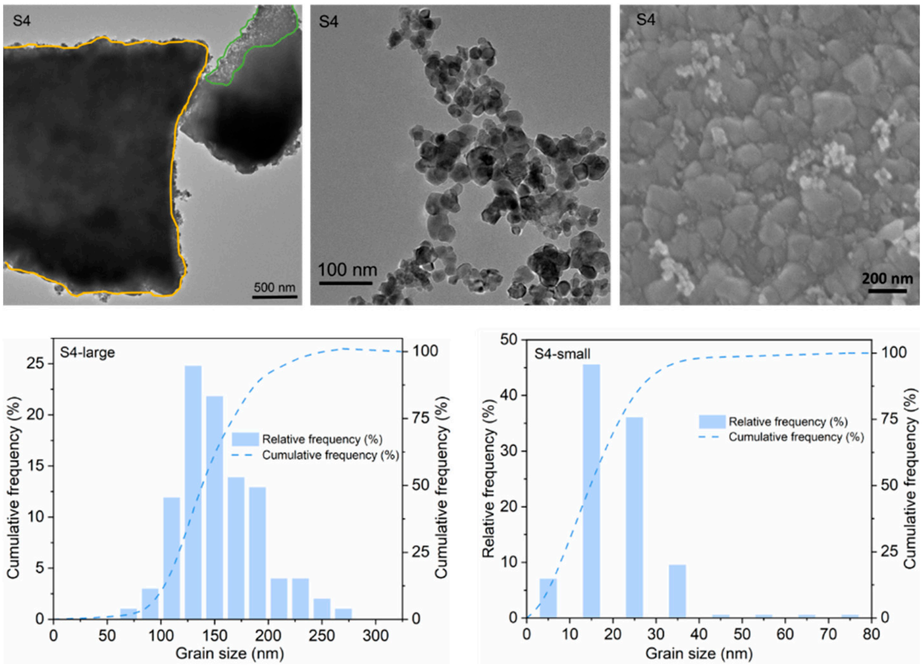

Figure S5. TEM, SEM images and corresponding grain size analysis of S4.

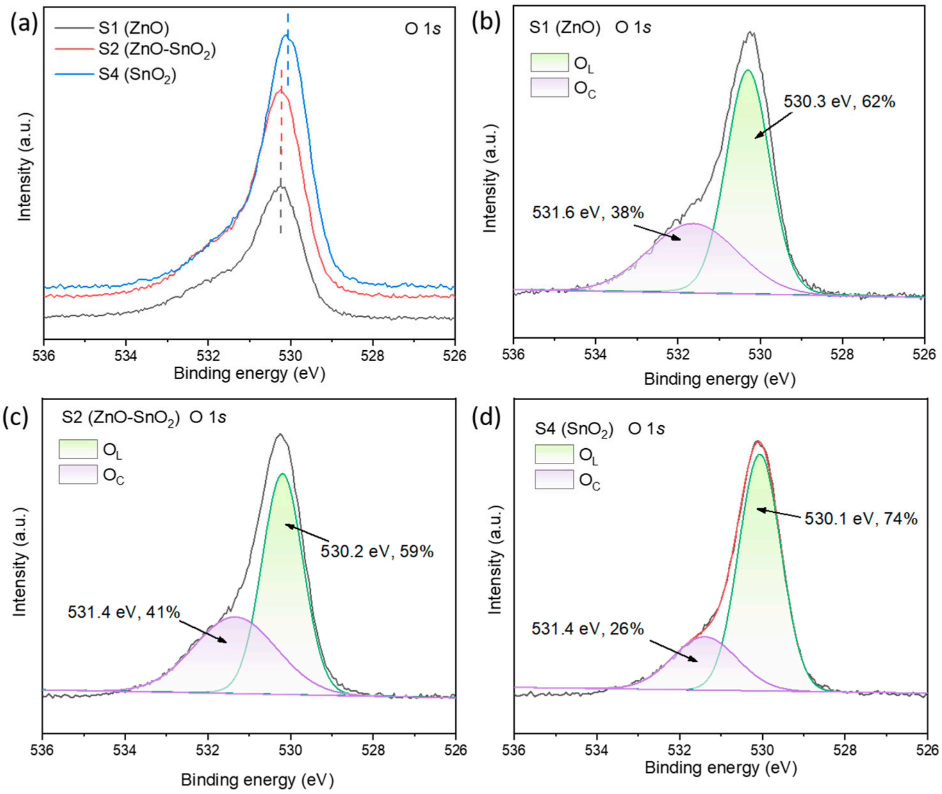

Figure S6. XPS curve of (**a**) O 1s for sample S1, S2 and S4; (**b**)-(**d**) O 1s peak area fitting results of S1, S2 and S4.

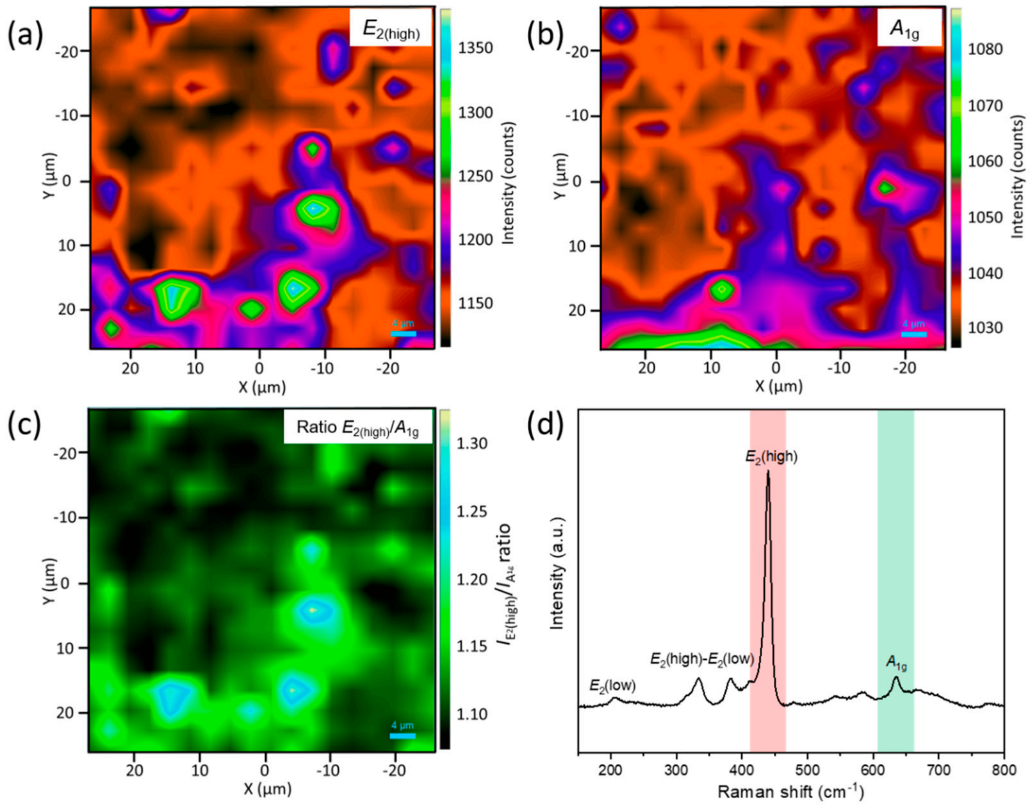

Figure S7. Raman mapping and spectra of ZnO-SnO_2. The intensity of Raman peak (**a**) $E_{2(high)}$, (**b**) A_{1g}. (**c**) $I_{E2(high)}/I_{A1g}$. (**d**) Raman spectra of ZnO-SnO_2.

The intensity of Raman spectroscopy can be used to quantitative analysis the amount, degree of crystallinity and distribution of different phases in the material. The peak intensity located around 439 cm^{-1} and 635 cm^{-1}, assigned to $E_{2(high)}$ and A_{1g}, which are the characteristic vibration modes of ZnO and SnO_2, respectively. The distribution of the $I_{E2(high)}/I_{A1g}$ ratio represents the distribution of different phase at the selected point, providing the spatial information of the two phases in micro level.

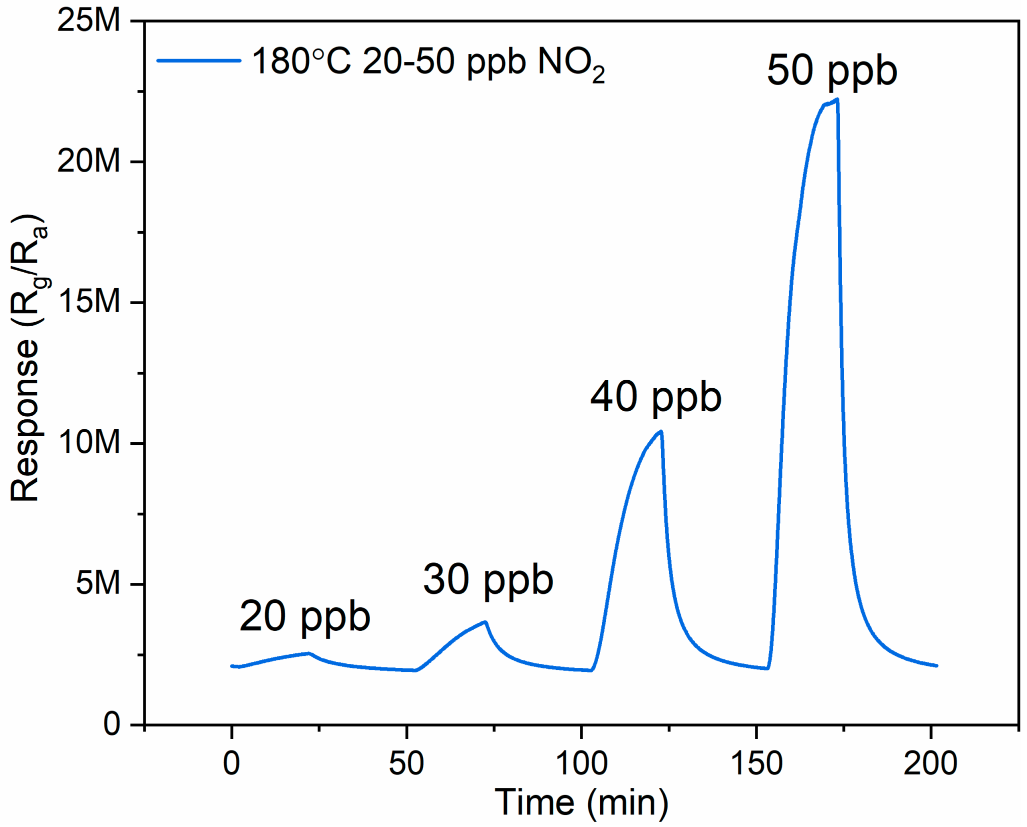

Figure S8. The real time resistance of sample S2 towards 20-50 ppb NO_2.

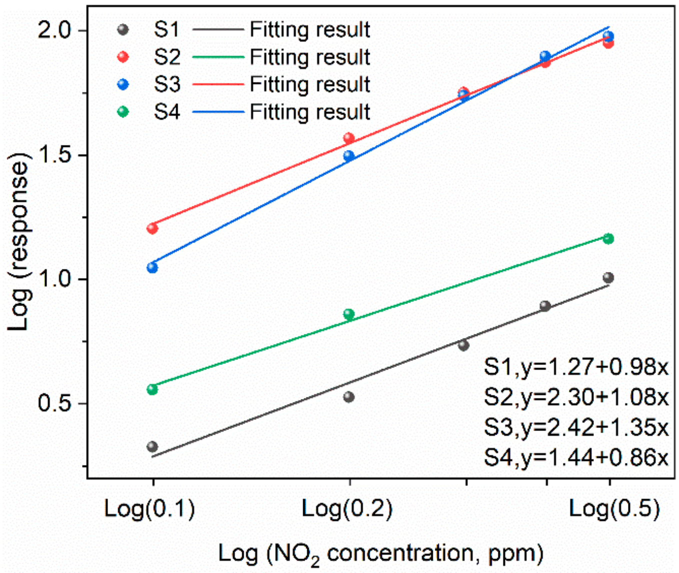

Figure S9. The correlation between response value and NO_2 concentration and the fitting curve.
